# The cellular uptake of angiogenin, an angiogenic and neurotrophic factor is through multiple pathways and largely dynamin independent

**DOI:** 10.1371/journal.pone.0193302

**Published:** 2018-02-27

**Authors:** Ross Ferguson, Vasanta Subramanian

**Affiliations:** Department of Biology and Biochemistry, University of Bath, Bath, United Kingdom; University of Edinburgh, UNITED KINGDOM

## Abstract

Angiogenin (ANG), a member of the RNase superfamily (also known as RNase 5) has neurotrophic, neuroprotective and angiogenic activities. Recently it has also been shown to be important in stem cell homeostasis. Mutations in *ANG* are associated with neurodegenerative diseases such as Amyotrophic Lateral Sclerosis (ALS) and Fronto-temporal dementia (FTD). ANG is a secreted protein which is taken up by cells and translocated to the nucleus. However, the import pathway/s through which ANG is taken up is/are still largely unclear. We have characterised the uptake of ANG in neuronal, astrocytic and microglial cell lines as well as primary neurons and astrocytes using pharmacological agents as well as dominant negative dynamin and Rab5 to perturb uptake and intracellular trafficking. We find that uptake of ANG is largely clathrin/dynamin independent and microtubule depolymerisation has a marginal effect. Perturbation of membrane ruffling and macropinocytosis significantly inhibited ANG uptake suggesting an uptake mechanism similar to RNase A. Our findings shed light on why mutations which do not overtly affect RNase activity but cause impaired localization are associated with neurodegenerative disease.

## Introduction

Angiogenin (ANG, also known as RNase 5) is a member of RNase A superfamily with a weak ribonucleolytic activity. The RNAse A superfamily comprises 8 canonical members [1], which includes the pancreatic ribonuclease (RNase 1 or A), eosinophil-derived neurotoxin (or RNase 2), eosinophil cationic protein (or RNase 3), RNase 4, angiogenin (ANG or RNase 5), RNase 6 (or k6), RNase 7, and RNase 8. ANG has a characteristic “CKXXNTF” signature motif, the catalytic triad, and six conserved cysteine residues and a signal peptide. Although its identity to RNAse A at the amino acid level is only 33%, the overall three dimensional structure is similar to RNAse A [2].

Variants in ANG are associated with neurodegenerative diseases such as Amyotrophic Lateral Sclerosis (ALS) and Frontotemporal dementia (FTD) [3–6]. Some of these variants result in a loss or impairment of the weak ribonucleolytic activity which appears to be critical for the neuroprotective function of ANG [7]. Besides active site residues, ANG-ALS variants are also frequently found in the nuclear localization signal as well as in the signal sequence of the ANG pre-protein [3–6]. Secreted ANG is taken up by cells and has been shown to initiate stress granule formation through cleavage of tRNA to tRNA-derived stress induced RNA (tiRNA) [8,9] leading to neuroprotection. More recently, ANG has been shown to play an important role in haematopoietic stem progenitor cells (HSPCs) [10]. ANG secreted by the haematopoietic stem cell niche promotes the proliferation of myeloid progenitor cells and also maintains the quiescence of the HSPCs [10] thereby regulating haematopoiesis.

The function of ANG as secreted protein has been best studied in the context of angiogenesis, wherein a gradient of ANG produced by cells in response to hypoxic conditions results in the migration of endothelial cells, and the formation of capillaries, in order to restore normal oxygen levels in a tissue [11–13]. An important step in this process is the uptake of ANG by the target cell. We and others have shown that neurons also take up ANG from culture media and is neuroprotective under stress conditions such as hypoxia [5,14–16]. Skorupa et al [17] did not observe nuclear localization in astrocytes or uptake of ANG by primary motor neurons under any condition in contrast to our findings which show that embryonic stem cell derived neurons and neuroblastoma cells [15,16] do take up ANG from the culture medium.

Uptake of exogenous or secreted proteins by mammalian cells involves elaborate import pathways [18,19]. These pathways include (1) clathrin mediated uptake which is dynamin dependent (2) caveolin mediated endocytosis which is associated with lipid rafts and is also dynamin dependent (3) macropinocytosis which is dynamin independent (4) and other less well studied pathways which are not mediated by clathrin or caveolin [19].

The import pathways involved in the uptake of two members of the RNAse A superfamily, RNase A and ECP, have been studied in some detail. RNase A which has 33% sequence identity to ANG and structural similarity [2] has been shown to be taken up by multiple pathways including clathrin coated vesicles, interaction with heparin sulfate proteoglycans (HSPG) and macropinocytosis [20]. Based on their findings, Chao and Raines [20] suggest that RNAse A behaves like a cell penetrating peptide. ECP, which also interacts with HSPG, has been clearly shown to be taken up only through macropinosomes [21]. Onconase (Ranpirnase) on the other hand has been suggested to be endocytosed through a clathrin mediated pathway although no Onconase specific cell surface receptors have so far been identified [22–24].

ANG is taken up by sub-confluent endothelial cells as well as by stem cell derived neurons and neuroblastoma cells [15,16,25] and is translocated to the nucleus. Oddly, despite having a nuclear localisation signal, a study by Lixin et al [26] showed that ANG did not require importins or Ran for passage through the nuclear pore and suggest that it is actively retained in the nucleolus in a GTP dependent manner.

ANG is a cationic protein which has been shown to bind very strongly to heparin [27,28]. Removal of cell-surface HSPG either by heparinase or blocking it by binding an excess of bFGF, prevented ANG uptake in calf pulmonary artery endothelial (CPAE) cells, as did sequestering ANG with an excess of free heparin [25].

To date several different proteins have been suggested to be receptors for ANG. Hu et al [29] reported a 170kD cell surface protein as a receptor for ANG in endothelial cells as well as an endothelial cell surface smooth muscle-type a/actin (or an a-actin-like protein) [30]. Skorupa et al., [17] reported syndecan 4, a HSPG as a receptor for ANG in astroglia since they found that uptake of ANG is affected upon knockdown of syndecan 4 by shRNA. They also showed that ANG uptake by astrocytes is inhibited by dynasore, a small molecule dynamin inhibitor, although a positive control for the effect of dynasore such the inhibition of transferrin uptake was not included in their study.

Given the conflicting findings in literature with regard to the uptake of ANG, we have sought to better understand the mechanisms of uptake and intracellular trafficking of ANG by established neural cell lines, primary cortical neurons and primary astrocytes. We have used well-characterised small molecule inhibitors of dynamin, dominant negative Dynamin and Rab5, as well as inhibitors of other import pathways to study uptake. We have also studied the mechanisms of intracellular transport.

## Results

We initially studied the time course of ANG uptake in SH—SY5Y cells, a neuroblastoma cell line [31], by exposing them to ANG at concentrations reported in human serum ([32,33], see materials and methods) in serum free medium. ANG is taken up from the media by SH—SY5Y cells and is detectable in the nucleus in a speckled distribution as early as five minutes after its addition (S1A Fig). The accumulation of ANG in the nucleus increases steadily as seen by the increased intensity of staining with the highly specific 26-2F anti-angiogenin antibody [34] and levels off at four hours of incubation. At this time point most of the ANG has been translocated to the nucleus with lesser amounts seen in the cytoplasm (S1A Fig). However, in neurons, a small number of punctate vesicle-like structures can be seen in neurites which increases after one hour of uptake. We found that ANG staining is seen in the nucleus is predominantly in structures staining intensely with DAPI i.e. in heterochromatin. (S1A Fig).

**Fig 1:**
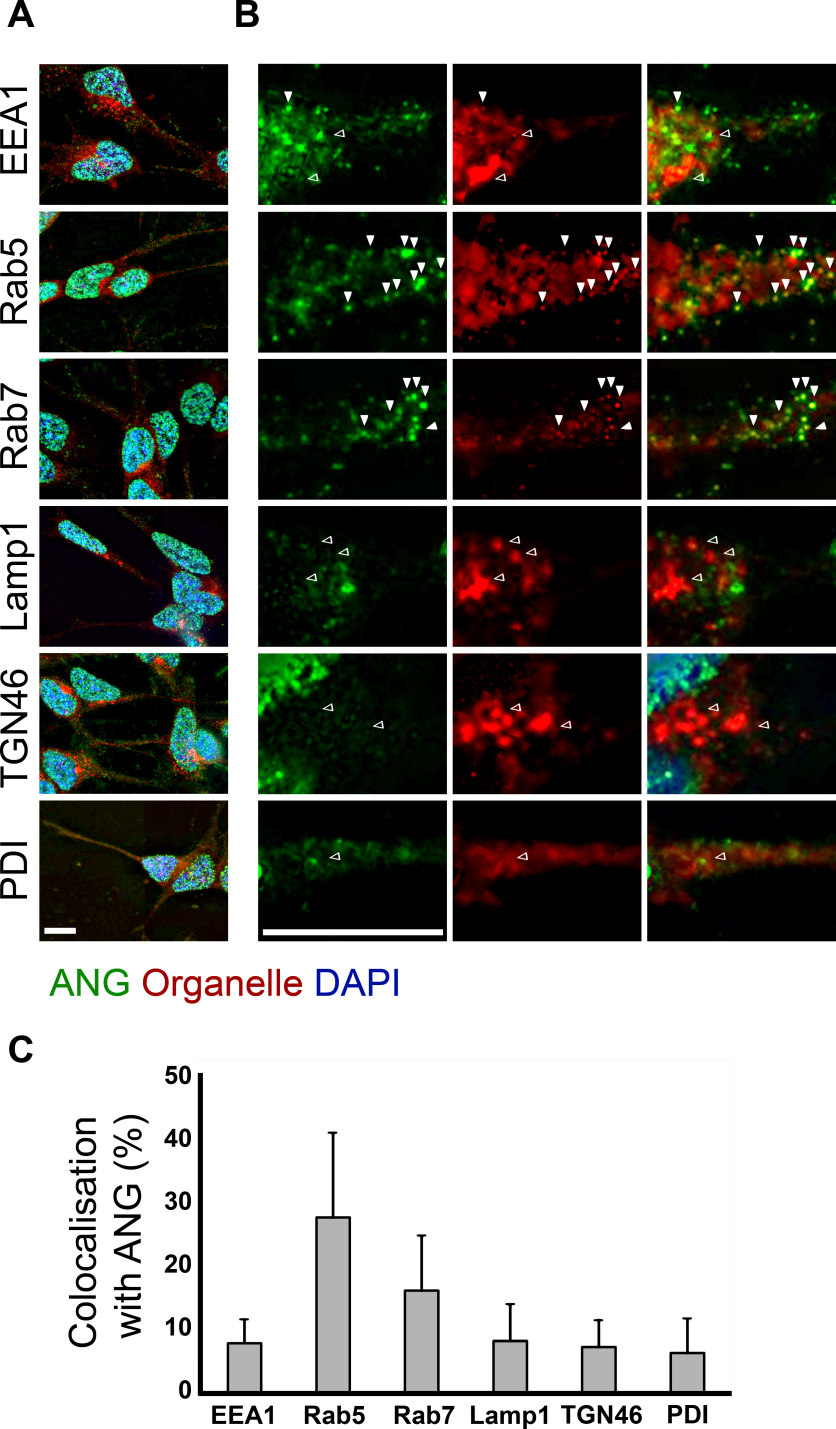
Co-localisation of ANG with organelle markers after uptake by SH-SY5Y cells. (A) SH-SY5Y cells were fixed after one hour of incubation with ANG (200ng/mL) and stained for ANG and markers of the various sub-cellular compartments. (B) The cytoplasm shows co-localisation of ANG with Rab5 and Rab7 positive compartments (white arrowheads arrows), ANG is seen less frequently in the EEA1 positive compartment and rare to no co-localisation with TGN46, Lamp1 and PDI (Black arrowheads). Scale bar: 10 μm. (C) For quantification of the proportion of the indicated organelle markers in the cytoplasm of SH-SY5Y cells that co-localise with ANG, at least ten cells were analysed individually, each from three independent experiments. Data is presented as the mean with standard deviation error bars.

**Fig 2:**
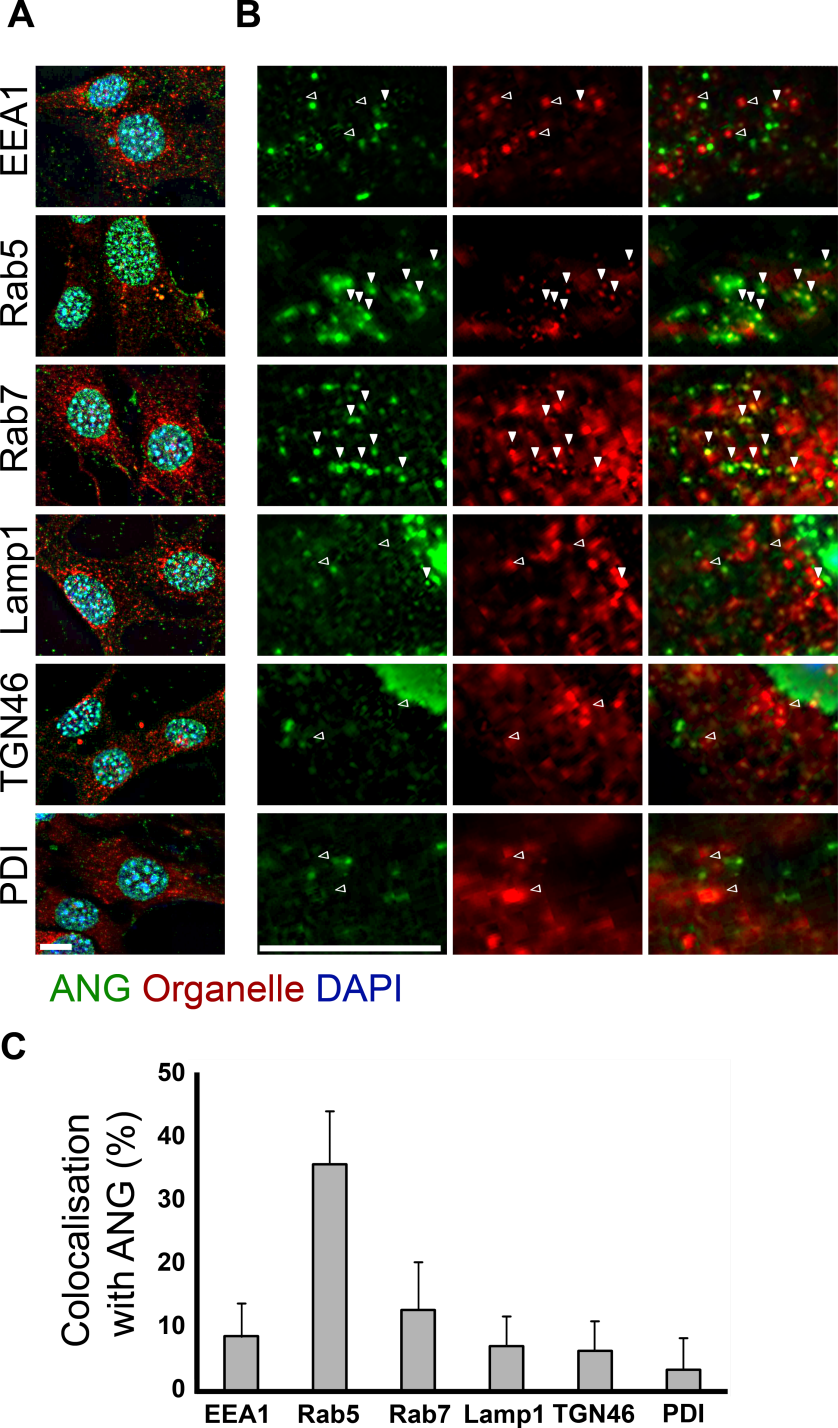
Co-localisation of ANG with organelle markers in C8-D1A. (A) After one hour of ANG uptake C8-D1A cells were fixed and stained for ANG and markers of the various sub-cellular compartments. ANG is present in the nucleus as well as the cytoplasm. (B) ANG localises in the Rab5 and Rab7 positive compartments (white arrows), is seen less frequently in the EEA1 positive compartment and rarely seen with TGN46, Lamp1 and PDI (Black arrows). Scale bar: 10 μm. (C) For quantification of the proportion of the indicated organelle markers in the cytoplasm of C8-D1A that co-localise with ANG, at least ten cells were analysed individually, each from three independent experiments. Data is presented as the mean with standard deviation error bars.

Co-immunostaining of ANG with organelle markers was performed to identify the sub-cellular compartments which contained ANG upon uptake. ANG can been seen to be present strongly in the nucleus as well as in the cytoplasm of both SH-SY5Y cells (a neuroblastoma cell line) and C8-D1A cells (an astrocytic cell line, [35]) after one hour of incubation (Fig 1A). Frequent overlap in staining can be seen in the cytoplasm with Rab5 positive compartments and more so with Rab7 in SH-SY5Y cells (Fig 1B, white arrows). ANG did not co-localise either with the early endosome marker EEA1 or with transferrin (Fig 1B). Similarly in C8-D1A cells, ANG was found to frequently co-localise with the Rab5 and Rab7 positive compartments in the cytoplasm, while overlap with EEA1 postive endosomes was very rare. (Fig 2B). ANG is also rarely seen in LAMP1 (lysosomal), TGN46 (Golgi) or PDI (endoplasmic reticulum) positive compartments in either SH-SY5Y or C8-D1A cells (Figs 1B and 2B, black arrows). Having established the time course of ANG uptake and cellular distribution of exogenously added ANG in SH-SY5Y cells, we investigated the effects of small molecule inhibitors of uptake and intracellular trafficking to identify the underlying mechanism. We also confirmed some of these findings by expressing dominant negative dynamin and Rab5.

### Effect of dynasore and Dyngo4a on the uptake of ANG by multiple cell lines

The large GTPase, dynamin plays a critical role in both the clathrin mediated as well as caveolin mediated endocytic pathways. Dynamin is essential for the fission of clathrin coated vesicles from the plasma membrane to generate endosomes and for the severing of caveolae from the plasma membrane. In order to determine whether the cellular uptake of ANG is clathrin mediated, we used two small molecule inhibitors of dynamin—dynasore and Dyngo4a and studied their effect on ANG uptake in (1) the neuroblastoma cell line SH-SY5Y; (2) the hybrid motor neuron cell line VSC4.1 [36]; (3) the astrocytic cell line C8-D1A [35]; (4) the microglial cell line BV2 [37] and (5) mouse primary cortical neurons and astrocytes. We quantified the staining intensity of ANG in the nucleus and cytoplasm. All uptake and drug treatments were carried out in serum free medium containing knockout serum replacement since serum contains ANG. To further support our data, we also perturbed clathrin mediated endocytosis using a dominant negative dynamin K44A and dominant negative Rab5.

There is emerging evidence that besides inhibiting the GTPase activity of dynamin, dynasore and Dyngo4a affect the labile cholesterol associated with the cell membrane thereby inhibiting the mobilization of the cell membrane and perturbing macropinocytosis [38,39]. It has also been shown that both dynasore and Dyngo4a inhibit fluid-phase endocytosis which can be clathrin mediated [38]. In our initial experiments we used dynasore but later on we also used a newer more potent inhibitor of dynamin, Dyngo4a [40]. In our ANG uptake experiments with dynamin inhibitors as well as with the dominant negative dynamin we used transferrin, a widely used marker for clathrin meditated endocytosis, as a control.

**Fig 3:**
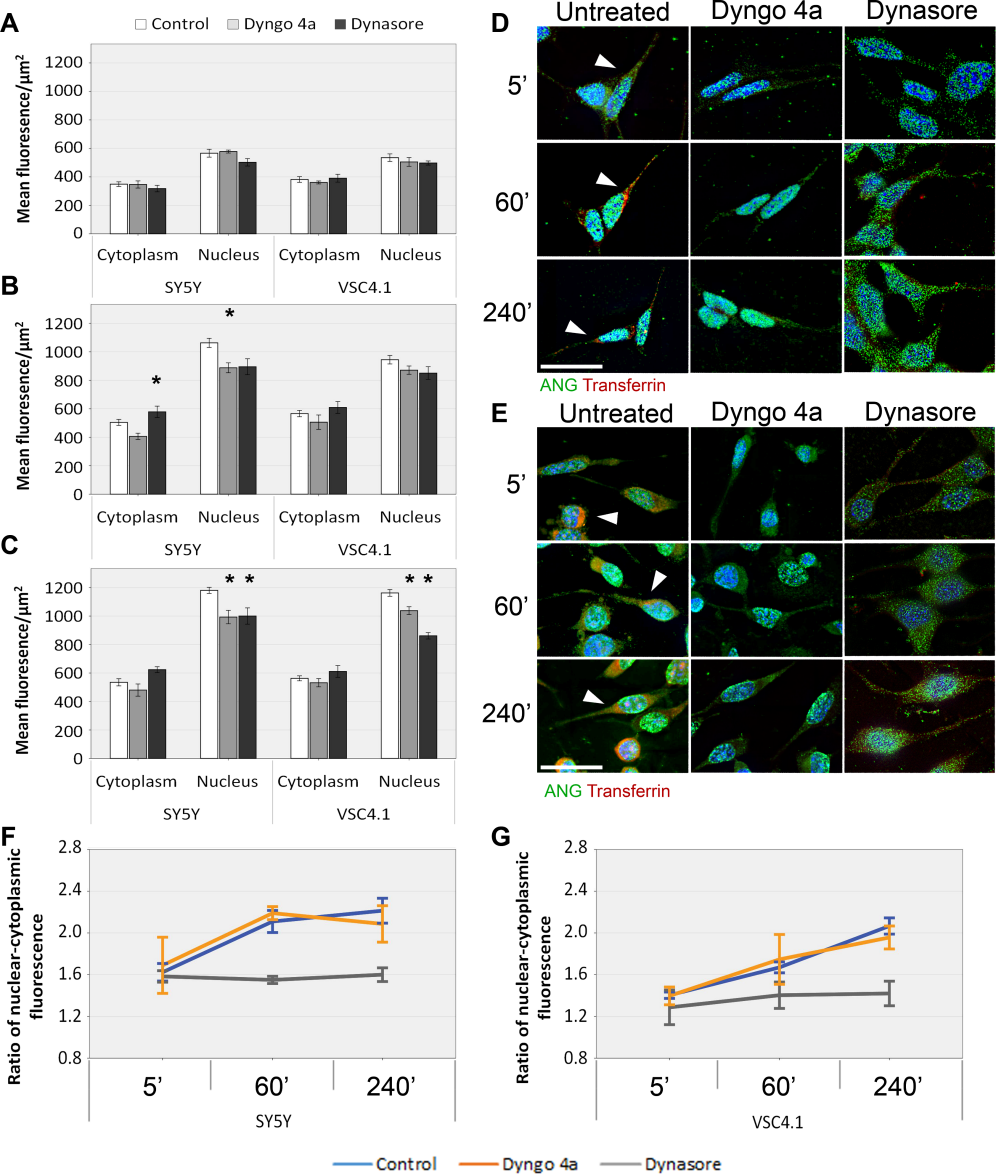
The effects of small molecule inhibitors of dynamin on the uptake of ANG by SH-SY5Y and VSC4.1 cells. SH-SY5Y and VSC 4.1 cells were pre-treated with either Dyngo4a or Dynasore for 30 minutes and then incubated with ANG in the presence of Dyngo4a or Dynasore and fixed and stained for ANG as described in Materials and Methods. ANG uptake by SH-SY5Y or VSC4.1 was quantified as mean fluorescence levels per square micrometre after five (A), sixty (B) and two hundred and forty minutes (C). Immunostaining of SH-SY5Y (D) and VSC4.1 (E) are shown for those time points and cells were also incubated with Alexa fluor 594 labelled transferrin as an uptake control. The ratio of nuclear to cytoplasmic mean fluorescence was calculated for both SH-SY5Y (F) and VSC4.1 (G) over the time course. Scale bar: 25 μm. Error bars SEM. The nucleus and cytoplasm of least ten cells were analysed from each of the three independent experiments performed. The mean fluorescence was compared by ANOVA, with Dunnett’s *post-hoc* comparison to the untreated control at each time point. n = 3, *P<0.05.

The uptake of ANG in VSC4.1 cells occurs rapidly and is detectable in the nucleus of untreated VSC4.1 cells as early as five minutes of incubation, as with SH-SY5Y cells (S1A Fig). Treatment with dynasore and Dyngo4a successfully blocked endocytosis of transferrin by SH-SY5Y and VSC4.1 cells but failed to prevent the uptake of ANG (Fig 3). No significant effect on the uptake of ANG in the presence of either Dyngo4a or dynasore was seen at five minutes. However, in dynasore treated cells we observed a striking effect on the cellular distribution of ANG with a weaker nuclear localisation and increased accumulation in the cytoplasm. Significantly less (p<0.05) ANG is found in the nucleus of SH-SY5Y after one hour of uptake in the presence of dynasore (Fig 3A–3G) concomitant with increased cytoplasmic ANG. After four hours of ANG uptake, the levels of nuclear ANG in dynasore treated cells was reduced by nearly 15% (p<0.05) (Fig 3C–3E) compared to the control. This effect is not seen in Dyngo4a treated cells, which while resulting in a small but significant (p<0.05) reduction in nuclear ANG in both cell lines, did not show increased cytoplasmic levels relative to untreated cells.(Fig 3C–3E).

The amount of ANG in the cytoplasm is not affected by Dyngo4a treatment at all three time points unlike in cells treated with dynasore (Fig 3D and 3E). When expressed as a ratio of nuclear to cytoplasmic ANG, untreated and Dyngo4a treated SH-SY5Y and VSC4.1 show an upward trend both at five minutes and one hour which then plateaus at four hours Fig 3F and 3G). This suggest rapid transport of ANG through the cytoplasm and translocation into the nucleus which reaches steady state at 4 hours to 1.5–2.0 fold higher level of ANG in the nucleus (Fig 3F and 3G). The nucleo-cytoplasmic ratio in dynasore treated SH-SY5Y and VSC4.1 showed no substantial change from five minutes to four hours with a ratio close to one, indicating a relatively even distribution of ANG throughout the cell suggesting an effect on nuclear retention.

In order to identify whether the effects of dynasore and Dyngo4a on ANG uptake by SH-SY5Y and VSC4.1 cells are peculiar to these cell lines, we performed these experiments with additional relevant cell types: C8-D1A, a type I astrocytic cell line, BV2, a microglia cell line as well as mouse primary cortical neurons and astrocytes.

The pattern of uptake of ANG by the microglial cell line BV2 and the astrocytic cell line C8-D1A are similar to that seen with SH-SY5Y and VSC4.1. ANG is present in the nucleus as early as five minutes after exposure in both cell lines (S2 Fig) with no significant differences in levels between the nucleus and cytoplasm (S2 Fig) upon treatment with Dyngo4a or dynasore. ANG levels in the nucleus increase after one hour of incubation in both BV2 and C8-D1A however significantly less ANG (p<0.05) is found in the nucleus of Dyngo4a treated cells, and this is more pronounced in dynasore treated cells (S2B Fig). Concomitant with the large reduction in in the amount of ANG seen in the nuclei of dynasore treated cells. A significant increase in cytosolic ANG is found in the cytoplasm of C8-D1A, this increase is not significant in BV2 cells however (S2F and S2G Fig). At four hours of incubation, ANG levels in the nucleus continue to increase in both BV2 and C8-D1A cells (S2C Fig) but significant reductions in nuclear ANG are seen in both cell lines treated with dynasore as compared to untreated cells. Dynasore treatment resulted in increased ANG levels in the cytoplasm of BV2 and C8-D1A cells, unlike untreated or Dyngo4a treated cells. The nuclear to cytoplasmic ratio of ANG increased and plateaued over the four hour time course in untreated and Dyngo4 treated BV2 and C8-D1A cells (S2F and S2G Fig). In contrast, dynasore treatment of these cells resulted in a nearly even distribution of ANG between the nucleus and cytoplasm at four hours i.e. less nuclear accumulation (S2F and S2G Fig).

**Fig 4:**
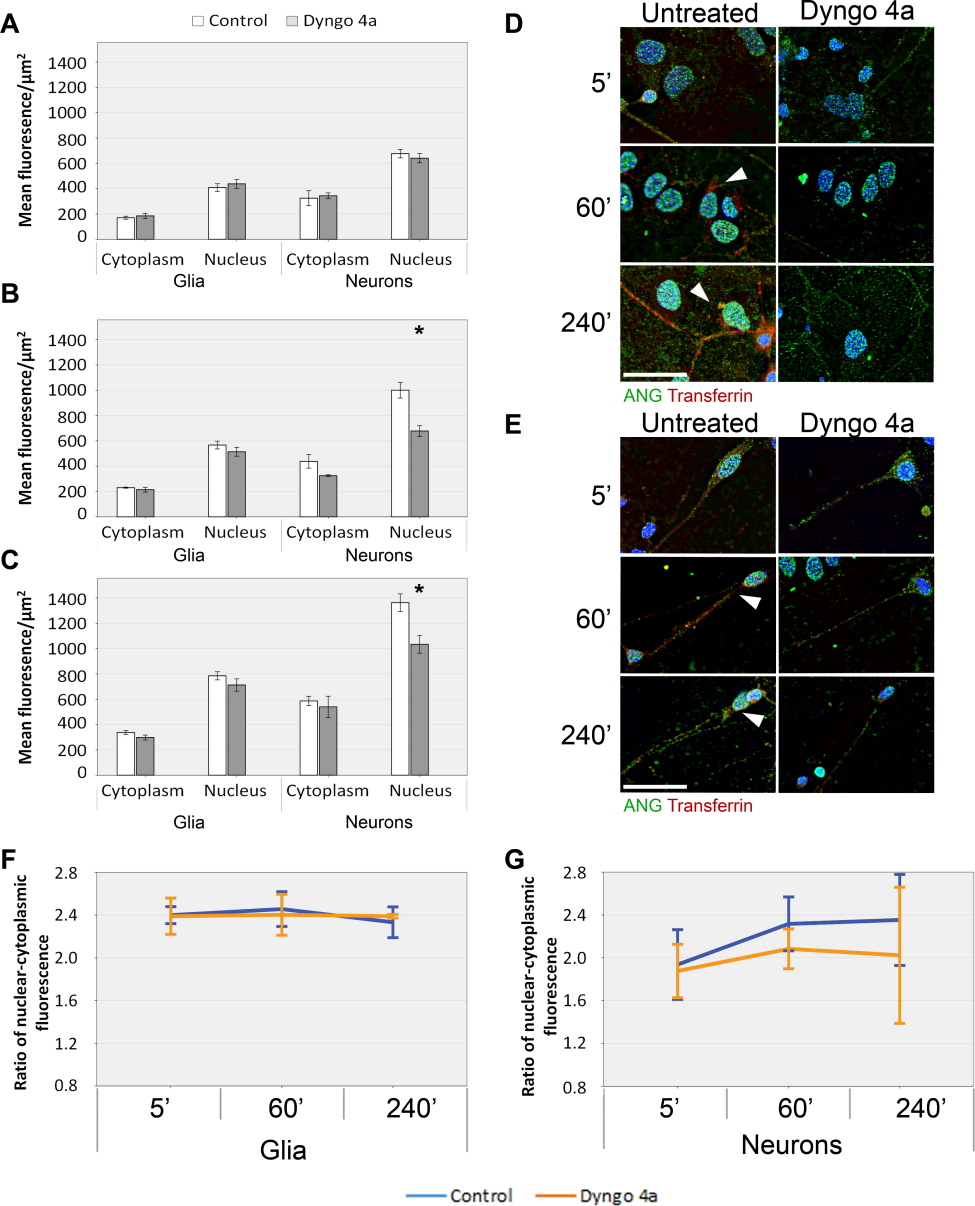
The effect of Dyngo4 on the uptake of ANG by primary cortical neurons and glia from P0 mouse cortex. Mixed cultures of primary cortical neurons and glia were pre-treated with Dyngo4a for 30 minutes and then incubated with ANG, fixed and immunostained for ANG. The uptake of ANG by neurons or glia was quantified as mean fluorescence levels per square micrometre after five (A), sixty (B) and two hundred and forty minutes (C). Immunostaining of glia (D) and neurons (E) are shown for the above time points. Cells both control and Dyngo4 treated were also incubated with Alexa fluor 594 labelled transferrin as an uptake control. The ratio of nuclear to cytoplasmic mean fluorescence was calculated for both glia (F) and neuron (G) over the time course. Scale bar: 25 μm. Error bars SEM. The nucleus and cytoplasm of least ten cells were analysed from each of the three independent experiments performed. The mean fluorescence was compared by t-test to the untreated control at each time point. n = 3, *P<0.05.

To assess whether the effect of Dyngo4A on ANG uptake in primary neurons and astrocytes is similar to that in established cell lines, we generated mixed cultures of primary neurons and glia from the cortex of P0 mice and performed ANG uptake experiments. As with the established cell lines, both primary neurons and glia show robust ANG uptake with ANG levels increasing in the nucleus over the four hour time course (Fig 4A–4E), Dyngo4a treatment resulted in significantly reduced ANG in the nucleus of neurons at one hour and four hours. The effect was more pronounced at one hour when the reduction in ANG levels was over 20% but this difference was reduced to only 5–10% by four hours (Fig 4D and 4E). The ratio of nuclear to cytoplasmic ANG in Dyngo4a treated primary neurons and glial cells is similar to the effects seen on the other cell lines (Fig 4F and 4G).

### Effect of dominant negative dynamin and Rab5 on endocytosis of ANG

We observed only small effects on ANG uptake by both primary and established cell lines using two small molecule inhibitors of the large GTPase dynamin. We sought to further confirm this using dominant negative approaches to perturb the clathrin pathway. The efficacy of transiently-expressed dominant-negative constructs to inhibit clathrin mediated uptake of ANG was confirmed by transferrin uptake. SH-SY5Y and C8-D1A cells expressing dominant negative dynamin do not take up transferrin while cells expressing dominant negative Rab5 appear to take up a small amount of transferrin which does not localise to the early endosome compartment (S3 Fig).

Besides the large GTPase dynamin, some members of the Rab family of small GTPase are also key regulators of the endocytosis. Rab5 is a critical mediator of Clathrin coated vesicle formation, endocytic internalization and early endosome fusion [41,42]. Having shown that the uptake of ANG is not greatly impaired by small molecule inhibitors of dynamin we sought to confirm this by expressing a dominant negative form of dynamin-dynamin K44A in the neuroblastoma cell line SH-SY5Y and the astrocytic cell line C8-D1A. We also investigated the effects of a dominant negative Rab5 in these two cell lines.

**Fig 5:**
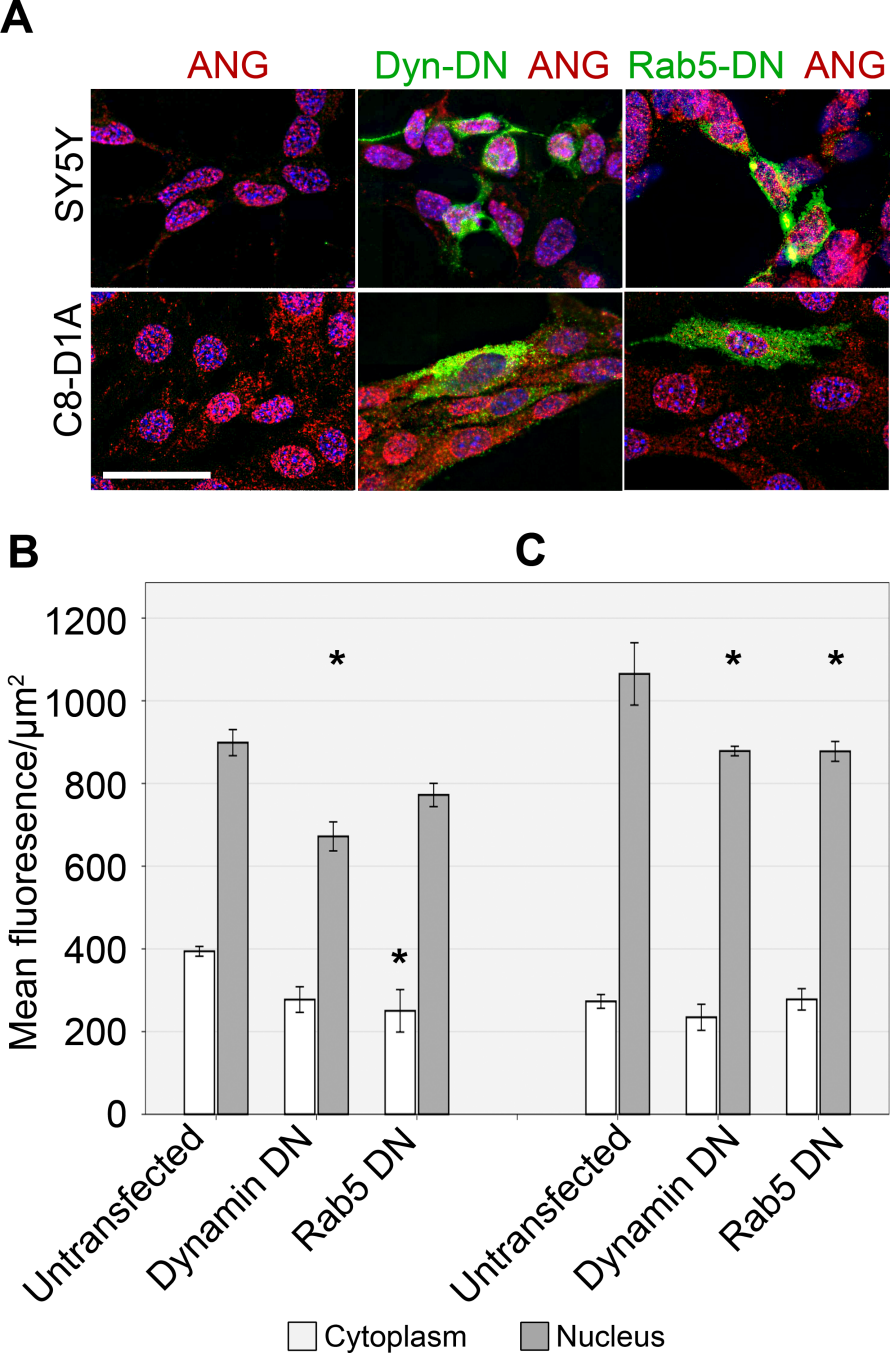
The effects of dominant negative Dynamin1 and Rab5 on ANG uptake by SH-SY5Y and C8-D1A cells. SY5Y and C8-D1A cell lines were transiently transfected with constructs encoding GFP tagged dominant negative Dynamin1 (K44A) or Rab5 (S43N). GFP was used to identify successfully transfected cells (A) 24h after transfection, ANG uptake was assessed by measuring mean fluorescence levels per square micrometre in the cytoplasm and nuclei of SH-SY5Y (B) or C8-D1A (C). Scale bar: 25 μm. Error bars SEM. The nucleus and cytoplasm of least twenty cells were analysed from each of the three independent experiments performed. The mean fluorescence was compared by ANOVA, with Dunnett’s *post-hoc* comparison to the untreated control at each time point. n = 3, *P<0.05.

ANG is taken up by untransfected SH-SY5Y and C8-D1A cells as well as by these two cell lines transfected with the dominant negative dynamin K44A construct (Fig 5). However, a small but significant reduction in ANG levels in the nucleus is seen in both SH-SY5Y and C8-D1A transiently transfected with a dominant negative dynamin K44A construct similar to that seen with dynasore treatment. A significant reduction in the nuclear levels of ANG was seen in C8-D1A cells expressing dominant negative Rab5 (S43N) (p<0.05). While the change in amount of nuclear ANG in SH-SY5Y was not significant (p = 0.053), significantly less ANG was seen in the cytoplasm of transfected cells (Fig 5). Dynamin K44A as well as dominant negative Rab5 inhibited the uptake of fluorescently labelled transferrin in the transiently transfected cells which were exposed to both ANG and transferrin (S3 Fig).

### Effect of inhibitors of macropinocytosis and lipid raft on ANG uptake by neuroblastoma and astrocytic cell lines

We observed small effects on ANG uptake by perturbing the clathrin mediated endocytosis. This led us to investigate if ANG, like ECP, is taken up by macropinocytosis or through lipid rafts. To investigate this we used three small molecule inhibitors in conjunction with ANG uptake by SH-SY5Y and C8-D1A cells—(1) Nystatin which disrupts lipid raft/ caveolae pathways (2) EIPA (5-(N-ethyl-N-isopropyl amiloride), an amiloride which is a potent and specific inhibitor of Na+/H+ exchanger activity which is essential for macropinosome formation (3) cytochalasin D which causes depolymerisation of F-actin leading to inhibition of membrane ruffling essential for the formation of macropinosomes. The uptake mechanisms of Transferrin, dextran and TAT containing peptides are well studied and they were used as controls to ensure drug efficacy (S1B Fig). Uptake of labelled dextran was used to monitor effect on fluid phase endocytosis, uptake of labelled TAT to monitor effect on macropinocytosis and uptake of labelled Transferrin to monitor effects on the caveolar pathway. In untreated SH-SY5Y and C8-D1A, Alexa Flour 594 labelled transferrin is seen in a perinuclear position in the early endosome after a one hour of uptake. Alexa Flour 594 labelled dextran is found in vesicles throughout the cytoplasm of both cell lines, as is a FAM labelled TAT containing peptide.

**Fig 6:**
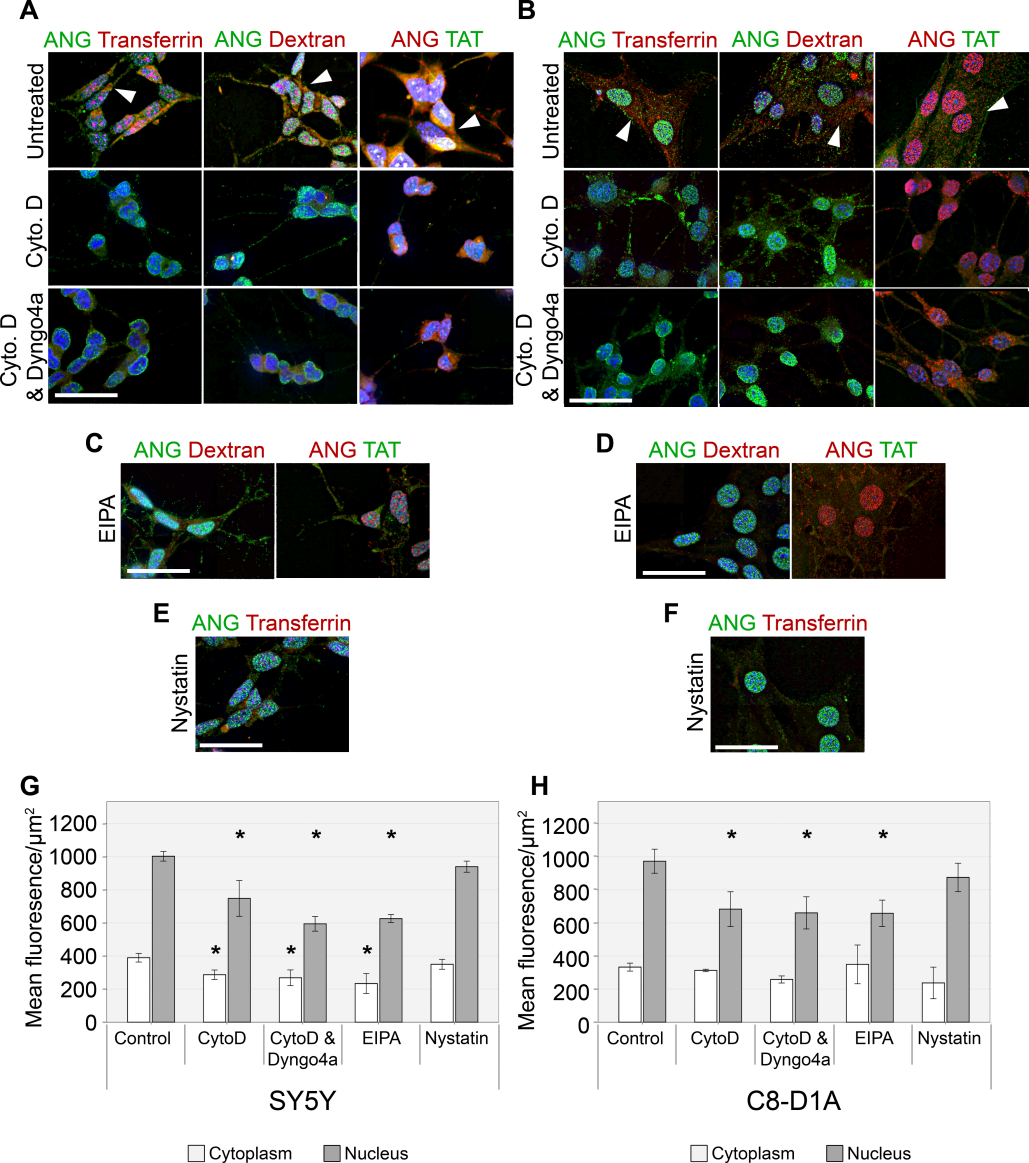
The effects of small molecule inhibitors on the uptake of ANG by SH-SY5Y and C8-D1A cells. SH-SY5Y (A) or C8-D1A (B) cells were incubated with ANG after pre-treatment with Cytochalasin D (Cyto. D) with or without Dyngo4a for 30 minutes, Similar treatments were performed with EIPA and Nystatin on ANG uptake by SH-SY5Y (C,E) and C8-D1A (D,F) cells. Cells were also incubated with Alexa fluor 594 labelled Transferrin or Dextran, and FAM labelled TAT peptide as uptake control. Mean fluorescence levels per square micrometre were determined in order to compare uptake between treatments (G,H). Scale bar: 25 μm. Error bars SEM. The nucleus and cytoplasm of least twenty cells were analysed from each of the three independent experiments performed. The mean fluorescence was compared by ANOVA, with Dunnett’s *post-hoc* comparison to the untreated control at each time point. n = 3, *P<0.05.

Uptake of ANG is significantly reduced after treatment with cytochalasin D, with significantly less seen in the nucleus after one hour of uptake (P<0.05, Fig 6). In both SH-SY5Y and C8-D1A cells treated with cytochalasin D, uptake of ANG and its nuclear accumulation was reduced by 20% (Fig 4G and 4H). The impairment of ANG uptake by cytochalasin D was enhanced in combination with Dyngo4A, leading to 25–30% decrease in ANG in the nucleus in both SH-SY5Y and C8-D1A cells (Fig 6). Significantly less ANG is found in the cytoplasm of SH-SY5Y cells after treatment with cytochalasin D and Dyngo4a when compared to untreated cells. There is no significant difference in the amount of cytoplasmic ANG in C8-D1A cells but a downward trend is observed between untreated to cytochalasin D alone or when cells are treated with a combination of cytochalasin D and Dyngo4a. ANG can be clearly seen accumulating in the plasma membrane of the neurites where the nucleus projects up from the flattened soma of SH-SY5Y after treatment with cytochalasin D (Fig 6A and 6B). This becomes more exaggerated in cells treated with cytochalasin D in combination with Dyngo4a but is only seen to a lesser degree in cells treated with Dyngo4a alone (Fig 6A and 6B, arrows). EIPA treatment also resulted in a 30% reduction in ANG accumulation in the nucleus in both SH-SY5Y and C8-D1A cells (Fig 6C, 6D, 6G and 6H) and leads to significant accumulation of ANG on the outer-membrane of the cell body, particularly in SH-SY5Y where it highlights small branches and dendrites otherwise unseen (Fig 6C and 6D). Treatment with Nystatin did not have significant effect on ANG levels in either the cytoplasm or the nucleus in both SH-SY5Y and C8-D1A cell lines Fig 6E and 6F). Treatment of the both cell lines with cytochalasin D and Dyngo4a prevents transferrin uptake, and considerably reduced the uptake of dextran but only modestly affected TAT uptake (Fig 6A and 6B). Similarly EIPA treatment of cells considerably reduced the uptake of dextran and modestly reduced TAT uptake (Fig 6C and 6D). Nystatin treatment of the cells prevents uptake of transferrin (Fig 6E and 6F).

### The effect of small molecule inhibitors of intracellular transport of angiogenin

Having established the uptake and the distribution pattern of exogenously added ANG in SH-SY5Y cells, we investigated the effects of small molecule inhibitors on the intracellular trafficking of ANG. The action of the small molecules was confirmed by characteristic redistribution of organelles identified by the specific markers PDI (ER), TGN46 (Golgi apparatus), RAB7 (late endosome), EEA1 (early endosome) and LAMP1 (lysosome).

**Fig 7:**
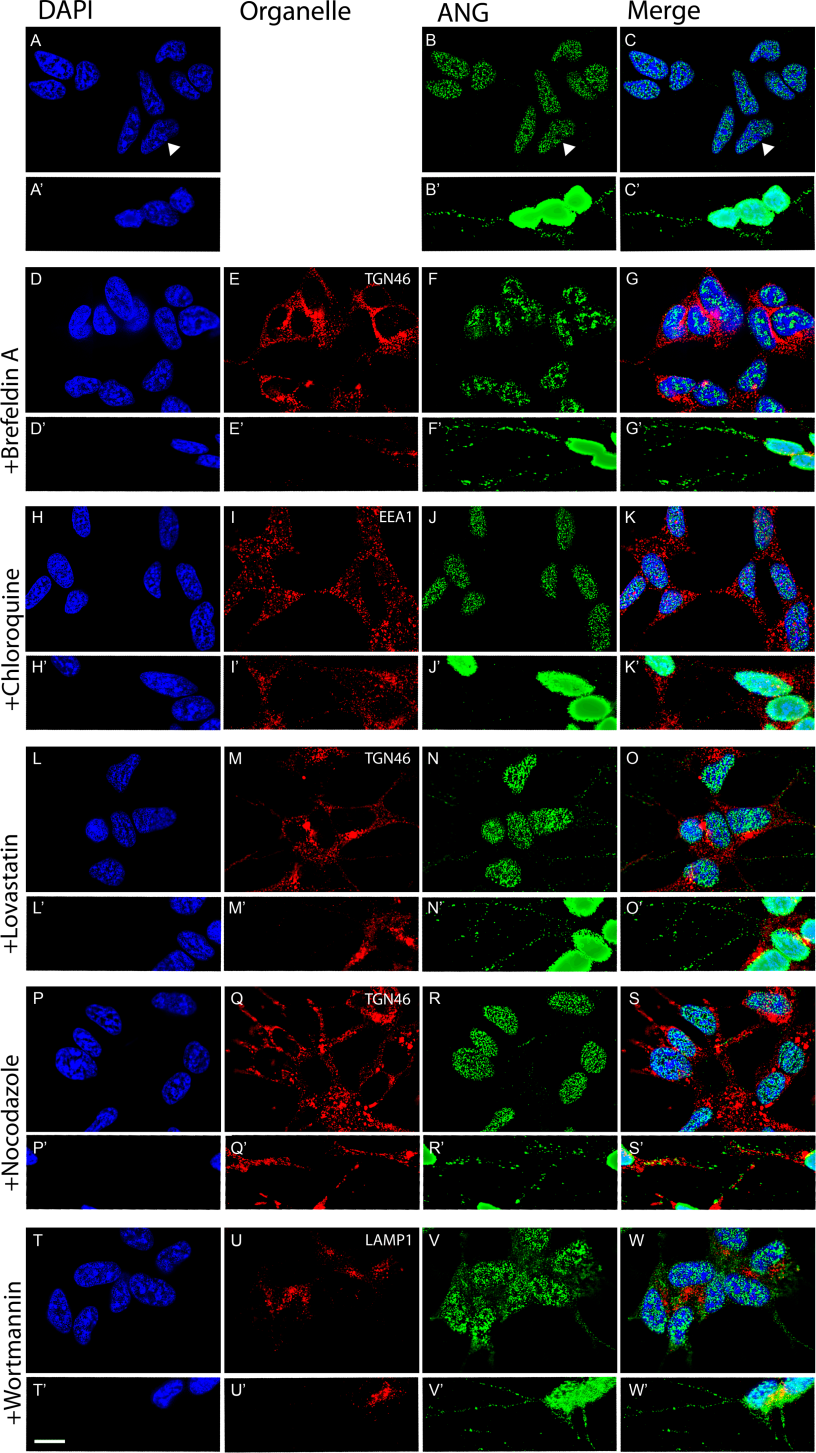
Effect of inhibitors of intracellular transport on the cellular distribution of angiogenin. In SH-SY5Y cells incubated with pure ANG protein there is rapidly translocation to the nucleus (a-c). After pre-treatment with small molecule inhibitors of intracellular transport, ANG still translocates to the nucleus but with altered intra-cytosolic and nuclear distribution. Small molecules used are Brefeldin A (d-g), Chloroquine (h-k), Lovastatin (l-o), Nocodazole (p-s) and Wortmannin (t-w). Panels labelled prime (‘) show a higher exposure image to highlight distribution within the neurite of the SH-SY5Y cells. Immunostaining for the drug-induced redistribution of the indicated organelle markers was used as an internal control. Scale bars for all images: 25μm.

In cells treated with Brefeldin A (BFA) disassembly of the Golgi stack occurs and redistribution to the ER takes place [43]. This occurs due to the inhibition of a guanidine nucleotide exchange factor preventing ARF and COPI mediated formation of transport vesicles between the ER and Golgi apparatus [44]. In BFA treated SH-SY5Y cells the TGN46 staining is redistributed throughout the cell but does not affect the uptake of ANG and its translocation to the nucleus. However, in BFA treated cells, distribution of ANG is less uniform with large aggregates in the cytoplasm which appear less evenly distributed compared to untreated SH-SY5Y cells. Increased number of ANG positive vesicle-like structures are seen in neurites where they appear to be larger and more frequent proximal to the cell body (Fig 7D’–7G’).

Chloroquine accumulates in the low pH compartments in the cell, preferentially in the lysosome. Accumulation results in inhibition of lysosomal enzymes due to increased pH, blocking transport to the lysosome [45,46]. Treatment of SY5Y with chloroquine results in characteristic swelling of the EEA1 positive early endosome due to impaired recycling (Fig 7I and 7I’). The uptake of ANG under these conditions by SH-SY5Y cells is unaffected. ANG accumulates in the nucleus in a pattern similar to untreated cells (Fig 7G and 7H) but appears to be reduced or absent in the cytoplasm and neurites (Fig 7H’–7K’).

Lovastatin is an inhibitor of 3-hydroxy-3-methylglutaryl-coenzyme A reductase (HMG-CoA reductase), a key component of the cholesterol biosynthesis pathway and causes a reduction of cholesterol levels in treated cells [47]. Lovastatin reduces cholesterol levels in the plasma membrane of treated cells leading to the disruption of lipid-raft dependent uptake. Lovastatin has been shown to inhibit the formation of both regulated and constitutive secretory vesicles. In SH-SY5Y cell treated with Lovastatin, the TGN46 staining compartment adjacent to the nucleus appears slightly condensed as compared with untreated cells (Fig 7M and 7M’). Lovastatin treated cells take up ANG and it is present in both the nucleus and neurites in a similar distribution and intensity to untreated cells (Fig 7L–7O and 7L’–7O’).

Intact microtubules appear to be required for rapid transport of ANG to the nucleus but not are not essential. Nocodazole disrupts microtubules by binding to β−tubulin and inhibits microtubule dynamics [48]. Neurites in SH-SY5Y treated with nocadazole are substantially shorter and stubby (Fig 7Q’). Redistribution of TGN46 is seen from its location adjacent to the microtubule organising centre to throughout the cell body, as expected upon dispersal of the TGN [49]. ANG is taken up by nocadazole treated cells and translocates to the nucleus (Fig 7P–7S) but its distribution in neurites appears as larger but fewer aggregates (Fig 7P’–7S’) as compared to untreated cells.

Wortmannin, a steroid metabolite is a non-specific, covalent inhibitor of phosphoinositide 3-kinases (PI3Ks). Due to this wortmannin has been shown to have an impact on multiple steps in uptake and intracellular trafficking, such as fluid-phase endocytosis, TGN to lysosomal transport and endosomal fusion (particularly rab5 dependant fusion in the early endosome) [50,51]. LAMP1 staining appears in slightly swollen compartments around the nucleus (Fig 7U and 7U’) in SH-SY5Y cells treated with wortmannin and staining for ANG appears slightly less intense in the nucleus but substantially more intense and evenly distributed throughout the cytoplasm (Fig 7T–7W). ANG distribution in the neurites appears similar to that in untreated cells (Fig 7T’–7W’).

### Uptake and nuclear translocation of ANG is essential for the activation of BV2 microglial cells

**Fig 8:**
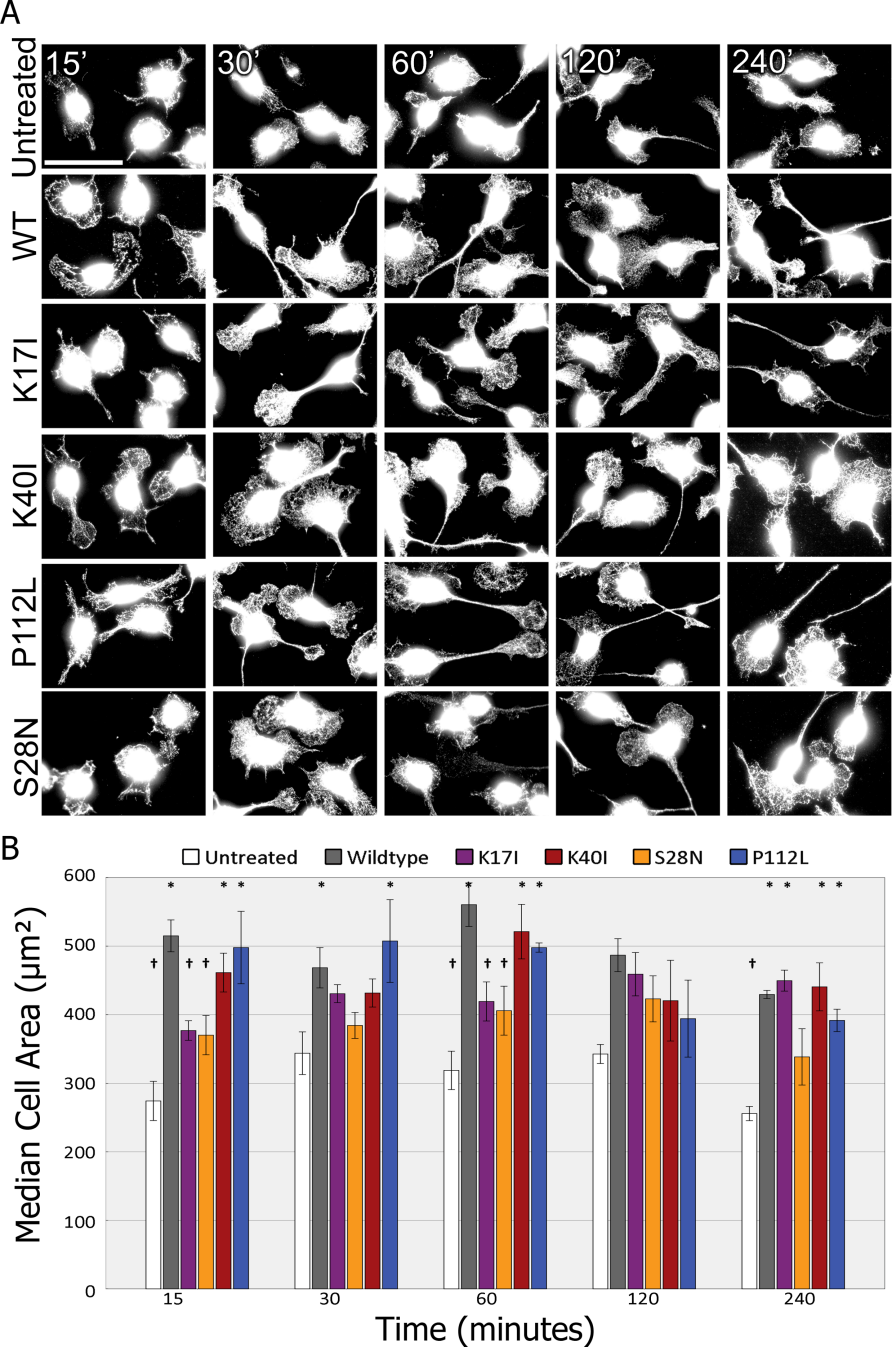
Nuclear translocation is required for ANG mediated BV2 microglia activation. A). BV2 cells treated with Dynamin inhibitors and angiogenin variants over a time course. Scale bars: 50μm. B) Median cell areas measured from six random fields from three independent experiments with 200–300 cells measured per experiment. Error bars SEM. The cell area was analysed from each of the three independent experiments performed. The mean of the natural log transformed data was compared by ANOVA, with Tukey’s *post-hoc*. n = 3, * P<0.05 between variant-treated and untreated BV2 cell area. † P<0.05 between variant-treated and untreated BV2 cell area.

BV2 cell incubated with dynasore and ANG show dramatic differences in morphology as compared to ANG alone. Control BV2 cells have very little cytoplasm with one or two thin short processes per cell (Fig 8). The addition of ANG results in a large increase in cytoplasmic area reminiscent of a phagocytic activated state. BV2 cells treated with both ANG and dynasore however fail to exhibit this change and are closer to untreated BV2 in morphology (S2 Fig). In addition to observing the effects of WT ANG, we used ALS associated variants with mutations we have previously shown to affect catalytic activity or trafficking [16]: K40I –Mutation of a basic lysine in the catalytic triad to aliphatic isoleucine results in a variant lacking ribonucleolytic activity (0.7%) while maintaining an unchanged 3d structure; P112L –Mutation of the cyclic proline to lysine results in large tertiary changes, also resulting in reduced catalytic activity (28.1%); K17I –Another catalytic triad residue mutation leaving only 13.1% activity and its proximity to the NLS results in impaired nuclear translocation; S28N –A mutation within the NLS resulting in impaired nuclear translocation and reduced ribonucleolytic activity (21.1%).

Untreated BV2 maintain median cell areas of 250–350μm^2^ throughout the course of the experiment. In contrast, during incubation with either WT ANG or ALS variants, the median area of BV2 cells increases over untreated cells (Fig 8). The largest increase in median cell area is seen with treatment with WT ANG at 15 and 60 minutes (505.18 μm^2^ (±23.19 SEM) and 579.49 μm^2^ (±31.75 SEM)). Treatment with ANG K17I results in a small increase in median cell area as seen with ANG S28N (350.91±28.63 μm^2^) after 15 minutes. While ANG K17I treated cell areas peak at 30 minutes (420.91±13.12 μm^2^), those treated with S28N show a slower response which peaks after 120 minutes (413.52±33.59 μm^2^). Both K40I and P112L treated cells follow a similar course peaking in area between 30 and 60 minutes (between 430–500 μm^2^) (Fig 8).

## Discussion

The uptake and nuclear translocation of ANG is critical for its function. Small molecule inhibitors of clathrin and caveolin mediated uptake, inhibitors of macropinocytosis and expression of dominant negative forms of Dynamin and Rab5 have allowed us to dissect the pathways through which ANG is taken up by different cell types of the central nervous system. ANG uptake and nuclear translocation appears to be very rapid and robust as none of the small molecule inhibitors or dominant negative forms of dynamin or Rab5 are able to completely inhibit uptake and nuclear translocation.

We found that ANG is taken up incredibly rapidly by SH-SY5Y and all other cell lines we have studied. This is unlike the observations by Moroianu and Riordan [25] who reported ANG in the cytoplasm after 5min and nuclear ANG after 15min in CPAE cells. We used ANG at levels found in serum and cerebro-spinal fluid and although this is only one fifth of the concentration of ANG used by Moroianu and Riordan, we observed uptake and nuclear translocation of ANG as early as 5min after addition. This difference may due to differences in the frequency of uptake sites on the membrane in each cell line.

It is curious that ANG colocalises with a proportion of Rab5 endosome but not EEA1 or Tfn. [52] show Rab5 colocalisation with APPL1/2 in EEA1 negative compartments in proximity to the plasma membrane. Interestingly they also show another (admittedly receptor mediated) angiogenic cytokine, EGF is internalised in APPL positive structures but not Tfn. Interestingly although dynamin II K44A overexpression blocked the classical translocation of EGF into the early and late endosomes, but EGF could still be seen in sub-membrane compartments. While a proportion of APPL1 endosomes have been shown to convert to EEA1 endosomes either directly or via a PI(3)P and WDFY2 intermediates, the fate of nearly half of the APPL1 endosomes is unclear [52]. In our case, perhaps the Rab5 colocalisation represents a proportion of the ANG taken up via dynamin. In an interesting parallel to ANG, APPL1 has been shown to shuttle from the cytoplasmic endosomal compartment to the nucleus where it associates with the nucleosome remodelling complex [52]. It is unclear how this nucleocytoplasmic shuttling occurs.

It is still unclear where ANG leaves the intracellular transport network. Recent studies have shown three populations of endo-lysosomal vesicles; Rab7-positive, Lamp1 positive and Rab7/Lamp1 positive, where Rab7/Lamp1 and Lamp1 only vesicles represent a terminal stage in dextran uptake [53,54]. We do not find ANG in Lamp1 positive compartments therefore it is not in lysosomes. Since we do not find ANG in the Lamp1 positive compartment, the Rab7 endosomes with which we do see colocalisation are unlikely to be terminal Rab7/Lamp1 endosomes but less mature Rab7 positive but Lamp1 negative endosome. This is emphasised by the differing location of the Rab7/ANG and Lamp1 stained populations: the Rab7 endosomes where we see colocalisation are within the bulk of the cytoplasm while the Lamp1 compartments are found much closer to the nuclear membrane. We do not know how the ANG population found in Rab5 positive EEA1 negative endosome moves to Rab7 positive, Lamp1 negative endosome, or how ANG leaves this pathway to reach the nucleus but our findings are consistent between the cell lines we have investigated. Dynasore and Dyngo4A are inhibitors of clathrin-mediated endocytosis which act by inhibiting the GTPase activity of dynamin 1 and 2 [55]. Dynasore and Dyngo4a were able to only block a very small proportion ANG from being taken up, indicating that ANG uptake is mostly independent of the clathrin mediated pathway. This is unlike another member of the RNase family, the cytotoxic Onconase which has been shown to be endocytosed through a clathrin mediated pathway. However, unlike ANG, Onconase transits to the late endosome/lysosome from where it enters the cytosol. However there are conflicting reports as to whether Onconase uptake is clathrin-mediated [23] or not [24]. Neither ANG, onconase nor other RNases co-localise with transferrin after endocytosis [22]. Skorupa et al., [17] have reported that ANG uptake in mouse astroglia is clathrin-mediated, while we show robust ANG uptake in astrocytic, microglial, neuroblastoma and hybrid motor neuron cell lines in the presence of inhibitors of dynamin which is a key player in clathrin-mediated endocytosis. As a control we show that the endocytosis of the transferrin receptor is affected in cells treated by dynasore and Dyngo4a. Additionally while we show classic nuclear ANG staining, Skorupa et al., [17] show ANG only in the cytoplasm despite using a five times higher concentration of ANG. Skorupa et al., [17] confirmed the observation by Moroianu and Riordan [25] that heparan sulfate proteoglycans are required for ANG uptake. Cellular uptake of proteins involving HSPGs has been reported to occur through multiple mechanisms including those which are clathrin and dynamin dependent depending on ligand and cellular context [56]. Another angiogenesis-related cytokine, FGF2 for example can enter the cell via clathrin-independent endocytosis mediated by both syndecan 4 and FGFR1 from lipid rich domains [57]. Alternatively the differences we see in the uptake of ANG from that of Skorupa et al [17] may be due the different to antibodies used to detect ANG. The antibody used in this study (26-2F) is highly specific to ANG and does not show cross-reactivity with other RNase A family members or angiogenin from other species [34]. Many alternative commercial clones we have tested have shown significant cross reactivity and poor specificity for ANG (Unpublished). Therefore the differences between our ANG localisation and uptake dynamics and those of Skorupa et al may also be partly be due to the use of different antibodies used in the immunostaining.

Dynasore treatment of cells failed to prevent uptake of ANG, but resulted in a reduced nuclear accumulation of ANG. Dynamin-like GTPases are involved in nuclear transport, for example the myxovirus resistance (Mx) proteins which are a family of dynamin-like GTPases. While MxA is expressed only in response to interferons, MxB is constitutively expressed. Knockdown of MxB did not impair nuclear import but expression of dominant negative forms (based on equivalent dynamin mutations) resulted in reduced import of a ~24kDa tagged nucleoplasmin [58]. Dynamin also has a role in the transport of cholesterol from the endo-lysosomal system and one known off target effect of dynasore is inhibition of vacuolar H+-ATPase preventing acidification of endosomes. As we have not found ANG in either of these compartments this appears to favour dynasore impairing the nuclear retention of ANG by inhibiting a dynamin or dynamin-like protein involved in nuclear transport such as the Mx GTPase family proteins, resulting in an equilibrium in ANG density between the nucleus and the punctate staining in the cytoplasm. This fits with observations made by Lixin et al., [26] that ANG passively enters the nucleus and is actively retained by a GTP-dependent mechanism independently of the conventional importin/RAN pathway. An alternative explanation may be that it is due to differing effects of Dyngo4a and dynasore on helical and ring assembled dynamin [40]. Short F-actin assemblies have been shown to induce the formation of ring dynamin, implicating it in actin dynamic in the cell [59]. This would fit with our observation that the actin depolymerising agent Cytochalasin D affects uptake but it is unclear how this would affect nucleo-cytoplasmic distribution.

We did not observe any endocytosed ANG in the Golgi or ER of SH-SY5Y, but disruption of COPI mediated transport by BFA treatment resulted in the presence of a much larger number of endocytic vesicles in treated SH-SY5Y. BFA has been shown to dramatically reduce endocytosis of heparan sulphate proteoglycans without affecting rates of clathrin-dependent or bulk endocytosis [60,61] thought to be due to the absence of essential components trapped at the ER. Another potential uptake pathway that may be blocked by BFA treatment would be the COPI-mediated Golgi to ER retrograde route. This route is required for the nuclear translocation of the EGF receptor (EGFR) from the cell membrane [62] and the transport of the simian virus 40 (SV40) to the ER [63]. Though it is unclear how ANG moves from endocytic vesicles to the nucleus this explanation seems unlikely as no accumulation is seen at the Golgi and ANG is not a membrane bound protein like EGFR. The increased number of vesicles seen in the neurites may simply be due to lower rates of vesicle turn-over in general due to the disruption of the TGN, this fits with the reduced, patchy nature of the nuclear ANG staining observed.

Intact microtubules appear to be required for rapid transport to the nucleus but not are not essential. After disruption of microtubules by nocodazole, ANG still successfully enters the nucleus confirming the observations of Li et al., [64] in endothelial cells. In addition, we see an effect of microtubule disruption on ANG-containing vesicles in the neurites; these appear as either multiple or fused larger vesicles bunched together with intervening large stretches of neurites lacking the large vesicles which are presumably trapped within the neurite. Without microtubules to transport ANG to the nucleus, transit within the cell must be occurring by other means. Multiple types of virus such as HIV1, Ad5, AcMNPV and adenoviral particles have been shown to utilise microtubules during their transit to the nucleus, yet nuclear translocation continues to occur after microtubule depolymerisation via the actin cytoskeleton [65–68].

We observed that neither chloroquine nor lovastatin had any effect on the transport of Angiogenin. Chloroquine has been shown to reduce endocytic recycling of the Alzheimer’s associated GM1 ganglioside in the brain, BMP receptor II in pulmonary endothelial cells and TGFβ receptor I in vascular endothelial cells by disrupting clathrin-mediated uptake [46,69,70]. Similarly lovastatin has been shown to inhibit clathrin-mediated endocytosis such as amyloid precursor protein and clathrin-independent proteins such as GPI-anchored Ecto-5'-Nucleotidase [71,72]. The mechanism by which chloroquine and lovastatin interfere with endocytosis is not well characterised. As lovastatin is a HMG CoA reductase inhibitor, depleted cholesterol resulting in the disruption of lipid-raft dependent uptake has been suggested as a mechanism by which endocytosis is blocked [73]. It has however been noted that lovastatin has little effect on overall cholesterol levels within cells by Ledoux et al., [71] who suggest a loss of actin remodelling by Rho-GTPases which require prenylation for their function may be the cause, as endocytosis could be rescued with the addition of a post-HMG CoA isoprenoid intermediate. It is unclear which mechanism is more relevant to ANG, if lipid rafts were affected one may expect reduced HSPG endocytosis (a proposed receptor of ANG) but lipid raft independent HSPG endocytosis has also been shown [56]. Similarly the mechanism by which chloroquine blocks clathrin-mediated endocytosis has been shown to be due to arrested maturation of the endocytic pit [74].

The location of ANG within the cell appears to be critical to its function. We have previously studied the dynamics of ANG uptake in SH—SY5Y cells and have shown that translocation to the nucleus is affected in some ALS associated ANG variants [16]. Two such variants, K17I and S28N show a retardation in nuclear translocation with a retention of ANG in the cytoplasm. Both these variants however retain 13.1% and 21.1% of WT RNase activity but the mutation affects the presentation of the NLS. Amongst other variants, P112L and K40I (28.1% and 0.7% activity) both show no changes to NLS structure and show strong nuclear translocation similar to that seen with WT ANG. Similar to the ANG-ALS variants in which the NLS is affected, SH-SH-SY5Y cells treated with Dynasore and incubated with wildtype ANG WT show a reduced nuclear import with increased cytoplasmic accumulation.

Our observations that treatment with ANG resulted in the activation of a microglial cell line are supported by the ability of ANG to activate Akt-1 [75], a pathway know to lead to microgila activation in response to lipopolysaccharides [76]. Activation of microglia was impaired when nuclear localisation of ANG was disrupted, either by dynasore or due to NLS-affecting mutations, implying ANG must reach its final destination before performing its function. It is interesting that the treatment of the BV2 microglial cell line with the structurally normal but ribonucleolytically inactive mutant ANG K40I resulted in a higher degree of BV2 activation than other mutants. ANG has been shown to induce nitric oxide synthase in endothelial cells independently of its RNase activity [11] therefore ANG K40I may be inducing activation of microglia through this alternative mechanism. [77]. Akt-1 is activated through PI3K which in turn is typically activated in a transmembrane fashion in response to a ligand binding. This would distinguish the nuclear role of ANG from the Akt-1 activating role since studies have shown that inhibition of PI3K prevents ANG-induced activation of Akt-1 without compromising nuclear translocation in endothelial cells [78]. A transmembrane signalling activity of ANG would mean the lack of catalytic activity in the K40I mutant would not be expected to compromise Akt-1 activation. This would explain its ability to activate the microglial cell line however Kieran et al [75] found that K40I failed to activate Akt-1 in NSC34 cells while Peng et al [79] show that ANG H37A activates Akt-1 in a bladder cancer cell line at similar levels to WT ANG. Again these differences may be cell type specific. The ALS-associated RNA metabolism proteins TDP43 and FUS both shuttle out of the nucleus in response to stress [80,81], indicating the nuclear or cytoplasmic localisation is a common critical control step though both these proteins are much larger than ANG and not necessarily imported/exported by the same mechanism.

In summary, ANG does not pass through the early or late endosomes upon uptake by cells which is largely clathrin/dynamin independent. Transit from the membrane occurs at a very rapid rate when microtubules are intact however they do not appear to be essential for nuclear translocation. Intracellular transport rate is also COPI sensitive but no Golgi to ER retrograde transport is observed. Trafficking into the nucleus itself is dynasore-sensitive and potentially dynamin-like dependent. Successful translocation to the nucleus is essential for inducing changes in microglia morphology associated with inflammatory activation. ANG uptake appears to occur using a combination of methods characterised in other pathways. Endocytosis at the membrane appears similar to other angiogenic molecules such as FGF and the subsequent route taken parallels other RNase family members such as RNAse A [20]. The mechanism by which translocation into the nucleus occurs and where ANG finally acts within the cell remain to be resolved. This in combination with the apparent disconnect between the neuroprotective tRNA cleavage and the multi-faceted PI3K-Akt pathway activation make ANG a very complicated protein despite its very small size.

## Materials and methods

### ANG proteins

Pure ANG protein as well as ANG ALS variants which had been characterised as described in Thiyagarajan et al., (2012) were provided by Dr Ravi Acharya.

### Cell culture

SH-SY5Y cells were obtained from ECACC (Porton Down, UK) and maintained on 10cm dishes in DMEM:F12 (1:1) containing Glutamax supplemented with 15% FBS (Labtech) and 1% NEAA (Life Technologies). VSC4.1 hybrid motor neuron cell line (obtained from Professor Angela Vincent, IMM, Oxford) cells were cultured on dishes coated with poly-l-ornithine (PLO) (Sigma) in DMEM:F12 (1:1) containing Glutamax (Life Technologies) supplemented with 2% FBS, 1% N1 (Sigma) and 1% NEAA. BV2 (hybrid microglial cell line) and C8-D1A (astrocyte cell line) were cultured in DMEM containing Glutamax (Life Technologies) with 10%FBS and 1% NEAA. Each cell line was passaged at 70–80% confluence by trypsinisation (Life Technologies) and split at a ratio of 1:3.

### Isolation and culture of primary cortical neurons

CD1 pups were sacrificed by schedule 1 method at P0 [UK (Animal Procedures) Act 1986, approved by AWERB]. Brains were dissected out in cold HBSS with 1x sodium pyruvate (Sigma), 0.1% glucose (Sigma), 1x penicillin/streptomycin (Life Technologies) and 10mM HEPES (Lonza). The cerebral cortex was isolated, minced and incubated in dissection solution containing 20U/mL Papain (Sigma), 1mM L-Cysteine (Sigma) and 0.5mM EDTA (Life Technologies) at 37°C for 15min. After washing in DMEM:F12 (Life Technologies) with 10% FBS (Labtech), 1X pen/strep and 1x sodium pyruvate, the cortices were disassociated by trituration with a 5mL pipette then flame polished glass pipettes. Disassociated cells were seeded onto poly-L-lysine (Sigma) coated plates. After four hours plating media was changed for maintenance media comprised of Neurobasal A (Life Technologies) with 1x pen/strep, 1x Glutamax. 50% media changes were made every three days. 1μM AraC (Sigma) was included from day 3.

### ANG uptake and treatment of cells with small molecule inhibitors

Pure recombinant human ANG and ANG ALS variants used in the uptake studies were provided by Professor Ravi Acharya. The purity of the proteins was confirmed by Mass Spec analysis. Cell lines were seeded at a density of 10^5^ cells/cm^2^ on acid-washed coverslips (SLS) in wells of 24-well plates (BD Falcon) in complete culture medium appropriate for the cell line. Coverslips were coated with PLO (10μg/cm^2^) for culture of VSC4.1 cell line and laminin (2.5μg/cm^2^) for the culture of primary cortical neurons. After 24h of culture, cells were incubated with pure recombinant ANG (200ng/mL) for uptake studies. For effects on ANG uptake by inhibitors, treatments were performed in DMEM:F12 with 0.5% KOSR (Life Technologies) at the following inhibitor concentrations: 80μM Dynasore (Abcam), 50μM Dyngo4a (Abcam), 5 μg/mL Cytochalasin D (Sigma), 10μM EIPA (Sigma), 25μg/mL nystatin (Sigma) 18mM Brefeldin A (Sigma), 0.1mM Chloroquine (Sigma), 0.1μM Wortmannin (Sigma), 1mM Lovastatin (Sigma) and 17mM Nocodazole (Sigma). Cells were pre-incubated for 2h with the appropriate inhibitor followed by both inhibitor and 200ng/mLANG protein in serum free DMEM:F12 with 0.5% KOSR. Alexa Fluor 568-conjugated Transferrin (25μg/mL), Alexa Fluor 568-conjugated dextran and a FAM-labelled TAT-peptide (1μM, Fl-TAT-4545W) were also included during the incubations with ANG as uptake controls. Cells were fixed for 15min in ice-cold methanol:acetic acid (3:1), washed twice with 70% ethanol and stored in 70% ethanol until used for immunostaining.

Since the highly specific Anti-ANG antibody 26-2F (kind gift of Dr Guo-Fu Hu) used to detect pure human ANG during uptake studies only works in Methanol fixed material, organelle specific marker antibodies were tested in both PFA and Methanol fixed cells to ensure consistency (data not shown).

### Transient transfection

GFP-Rab5 N113I, pCMV myc-rEpsin1 R62L H73L (kind gifts from Giampietro Schiavo), pEGFPN1-human Dynamin 1aa K44A was a gift from Pietro De Camilli (Addgene plasmid # 22197, Lee and Camilli, 2002) and GFP-rab7 DN was a gift from Richard Pagano (Addgene plasmid # 12660, Choudhury et al., 2002). Plasmids were prepared using Promega Wizard® Plus Maxiprep kit following manufacturer’s instructions. Transient transfections were performed on semi-confluent cells using Fugene HD (Promega) at a DNA to reagent ratio of 1:3 following manufacturer’s instructions. Uptake of ANG by the transfected cells were performed as described above in—**ANG uptake and Treatment of cells with small molecule inhibitors** at 24 and 48 hours post-transfection.

### Immunocytochemistry

Inhibitor treated cells and controls stored in 70% ethanol were rehydrated to PBS before incubation at room temperature for one hour in blocking solution (0.1% gelatin, 1% FBS, 0.5% Triton X-100 (BDH) in PBS). Cells on were incubated overnight at 4°C with primary antibodies diluted in blocking buffer. Non-specific antibody binding was removed by four 10min washes with PBS-T (0.1% Triton X-100 in PBS) followed by incubation for two hours with appropriate secondary antibodies and DAPI diluted in blocking buffer. Non-specific binding was removed by four 10min washes in PBS-T and two 10min PBS washes before mounting in Mowiol (Sigma). Immunostained cells were observed on a Leica DMRB microscope. Images were acquired using LAS-AF (Leica) and Figs composed in Photoshop (Adobe). For antibody details see Table 1.

**Table 1:** Primary antibodies used for immunostaining.

Antibody	Dilution	Supplier	Cat#
EEA1	1:200	Cell Signal Technologies	#3288
GFP	1:500	Thermo Fisher	A-11122
Human Angiogenin	1:1000	A gift from Dr. Guo-Fu Hu	Clone 26-2F
LAMP1	1:500	Abcam	Ab24170
PDI	1:200	Cell Signal Technologies	#3501
PKC zeta	1:500	Abcam	Ab51157
RAB5	1:200	Cell Signal Technologies	#3547
RAB7	1:200	Cell Signal Technologies	#9367
TGN46	1:500	Abcam	Ab16052

### Quantification of ANG-organelle colocalisation

Organelle-ANG colocalisation was quantified using Fiji. The cytoplasm of each cell was segmented by hand, excluding the nucleus. The number of organelles in each cell was determined using the particle analyser on a binary mask created first by thresholding the organelle channel based on intensity, followed by watershed to split overlapped objects. The Colocalisation colourmap plugin (2010, A. Gorlewicz) was used to generate a mask showing overlapping pixels, this was thresholded and the watershedded binary image then quantified by particle analyser. The proportion of the total organelle population of each cell found to also contain ANG was expressed as a percentage and presented as a mean of the cells analysed. Three independent experiments were conducted with analysis performed on at least ten cells from each experiment using differing passage cell lines on different dates.

### Quantification of fluorescence intensity of angiogenin immunostaining in cells

Images of stained cells were acquired from at least 10 fields each containing a minimum of 1–2 cells each. Exposure was set for each cell line using the conditions giving the maximal uptake during the experiment (i.e. untreated cells incubated with Ang for four hours) to prevent the clipping of data and the same settings used for each image acquired from that cell line under different conditions. Images were taken as Z-stacks and deconvoluted prior to analysis.

Image analysis was performed in ImageJ [82]. Angiogenin staining was used to delineate the outer edge of the cells while DAPI staining identified nuclear regions. Both the area and the total fluorescence within these two regions (i.e. the cytoplasm and nucleus) was measured and converted into mean fluorescence per μm^2^.

Three independent experiments were performed using differing passage cell lines on different dates or cortical neurons from brains of independent mouse litters. Experimental means were compared by ANOVA followed by Dunnett’s *post hoc* comparison to the untreated control at each respective time point. All statistical analyses were performed in SPSS (IBM).

### Quantification of BV2 cell activation by angiogenin

BV2 cells were plated and after overnight culture were incubated with ANG variants then fixed at indicated time points as described in the previous sections. BV2 cells were immunostained for PKC zeta to visualise the whole cell easily. The area of BV2 cells was measured using imageJ (NIH). Median cell areas were calculated from cells in six random fields from three independent experiments with 200–300 cells per experiment. The data wastransformed to a normal distribution by natural log. Experimental means were compared by ANOVA followed by Tukey’s *post hoc* comparison at each respective time point. We have highlighted only the comparisons between either the untreated cells or those treated with wildtype ANG, see Fig 8 legend. All statistical analyses were performed in SPSS (IBM).

## Supporting information

S1 Fig Time course of uptake of ANG and uptake of endocytosis control molecules in multiple cell lines. (A) The cell lines SH-SY5Y, VSC4.1, BV2 and C8-D1A incubated with 200ng/ml ANG for the indicated time show up take as soon as five minutes after exposure with saturation in the nucleus between one and four hours. Scale bars 25μm. (B) Localisation of ANG and endocytosis control compounds after one hour of uptake in SH-SY5Y and C8-D1A. ANG shows no co-localisation with transferrin, but a small amount of co-localisation is seen with dextran in the cytoplasm and TAT in the nucleus and cytoplasm (white arrows). Very little overlap is seen with TAT found in the cytoplasm. Scale bars 10μm.
(TIF)

S2 Fig The effects of small molecule inhibitors of dynamin on the uptake of ANG in C8-D1A and BV2 cells. After pre-treatment with either Dyngo4a or Dynasore for 30 minutes, ANG uptake by C8-D1A or BV2 was quantified as mean fluorescence levels per square micrometre after five (A), sixty (B) and two hundred and forty minutes (C). Immunostaining of C8-D1A (D) and BV2 (E) are shown for those time points and cells were also incubated with Alexa fluor 594 labelled transferrin as an uptake control. The ratio of nuclear to cytoplasmic mean fluorescence was calculated for both C8-D1A (F) and BV2 (G) over the time course. Scale bar: 25 μm. The nucleus and cytoplasm of least ten cells were analysed from each of the three independent experiments performed. The mean fluorescence was compared by ANOVA, with Dunnett’s *post-hoc* comparison to the untreated control at each time point. N = 3, *P<0.05.
(TIF)

S3 Fig Dominant negative dynamin and Rab5 block transferrin uptake. Robust uptake of Alexa 594 labelled transferrin can be seen in both untransfected SH-SY5Y (A) and C8-D1A (B). Transient transfection with either GFP-tagged dominant negative Dynamin1 (Dyn DN) or dominant negative Rab5 (Rab5 DN) prevents transferrin uptake. Scale bars 10μm.
(TIF)
