# Supplementary figures and images for: The cellular uptake of angiogenin, an angiogenic and neurotrophic factor is through multiple pathways and largely dynamin independent
Source: PLoS One. 2018 Feb 27;13(2):e0193302. doi: 10.1371/journal.pone.0193302 (PMC5828446; doi:10.1371/journal.pone.0193302)

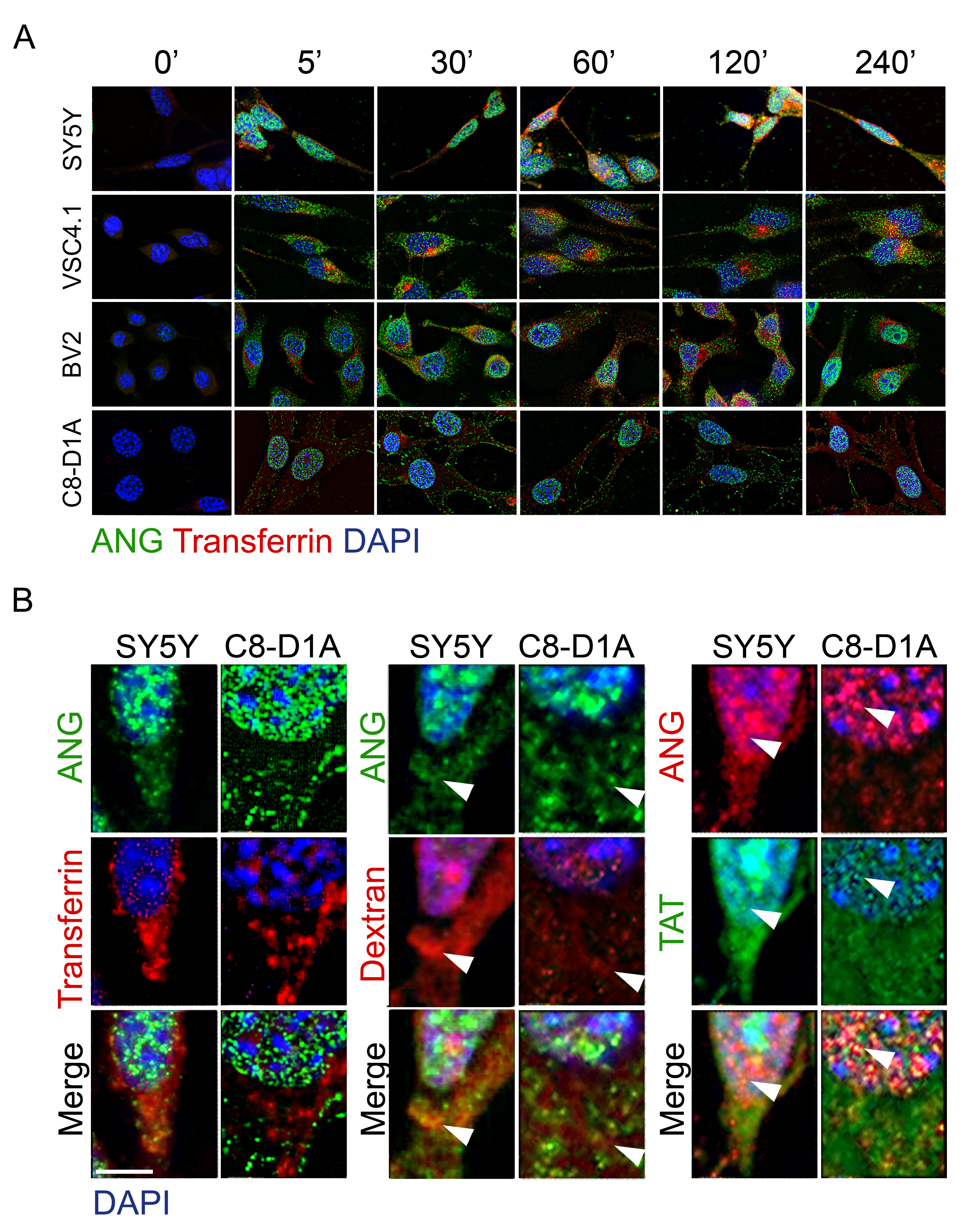

Supplement: S1 Fig — (A) The cell lines SH-SY5Y, VSC4.1, BV2 and C8-D1A incubated with 200ng/ml ANG for the indicated time show up take as soon as five minutes after exposure with saturation in the nucleus between one and four hours. Scale bars 25μm. (B) Localisation of ANG and endocytosis control compounds after one hour of uptake in SH-SY5Y and C8-D1A. ANG shows no co-localisation with transferrin, but a small amount of co-localisation is seen with dextran in the cytoplasm and TAT in the nucleus and cytoplasm (white arrows). Very little overlap is seen with TAT found in the cytoplasm. Scale bars 10μm. (TIF) [file pone.0193302.s001.tif]

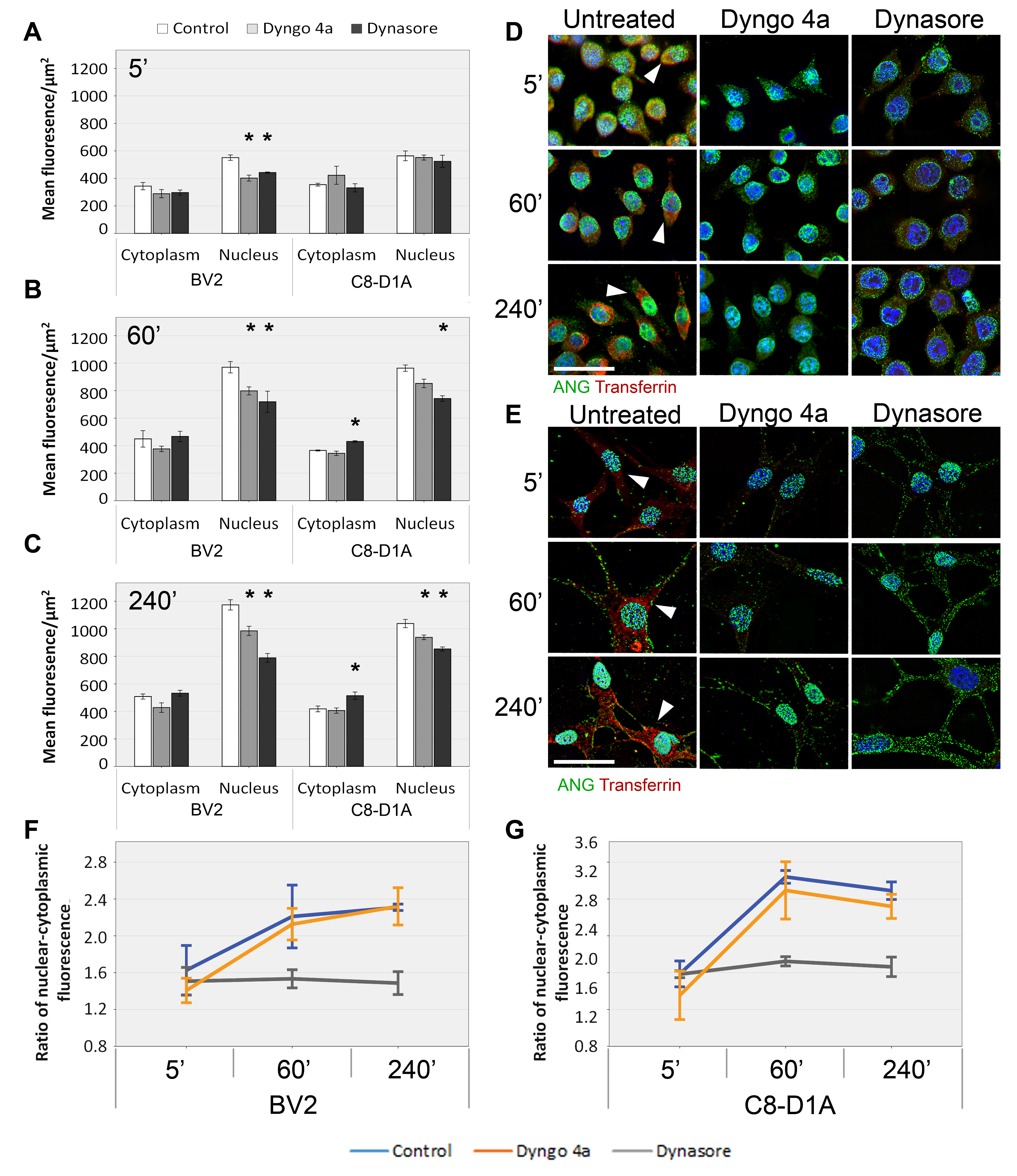

Supplement: S2 Fig — After pre-treatment with either Dyngo4a or Dynasore for 30 minutes, ANG uptake by C8-D1A or BV2 was quantified as mean fluorescence levels per square micrometre after five (A), sixty (B) and two hundred and forty minutes (C). Immunostaining of C8-D1A (D) and BV2 (E) are shown for those time points and cells were also incubated with Alexa fluor 594 labelled transferrin as an uptake control. The ratio of nuclear to cytoplasmic mean fluorescence was calculated for both C8-D1A (F) and BV2 (G) over the time course. Scale bar: 25 μm. The nucleus and cytoplasm of least ten cells were analysed from each of the three independent experiments performed. The mean fluorescence was compared by ANOVA, with Dunnett’s post-hoc comparison to the untreated control at each time point. N = 3, *P<0.05. (TIF) [file pone.0193302.s002.tif]

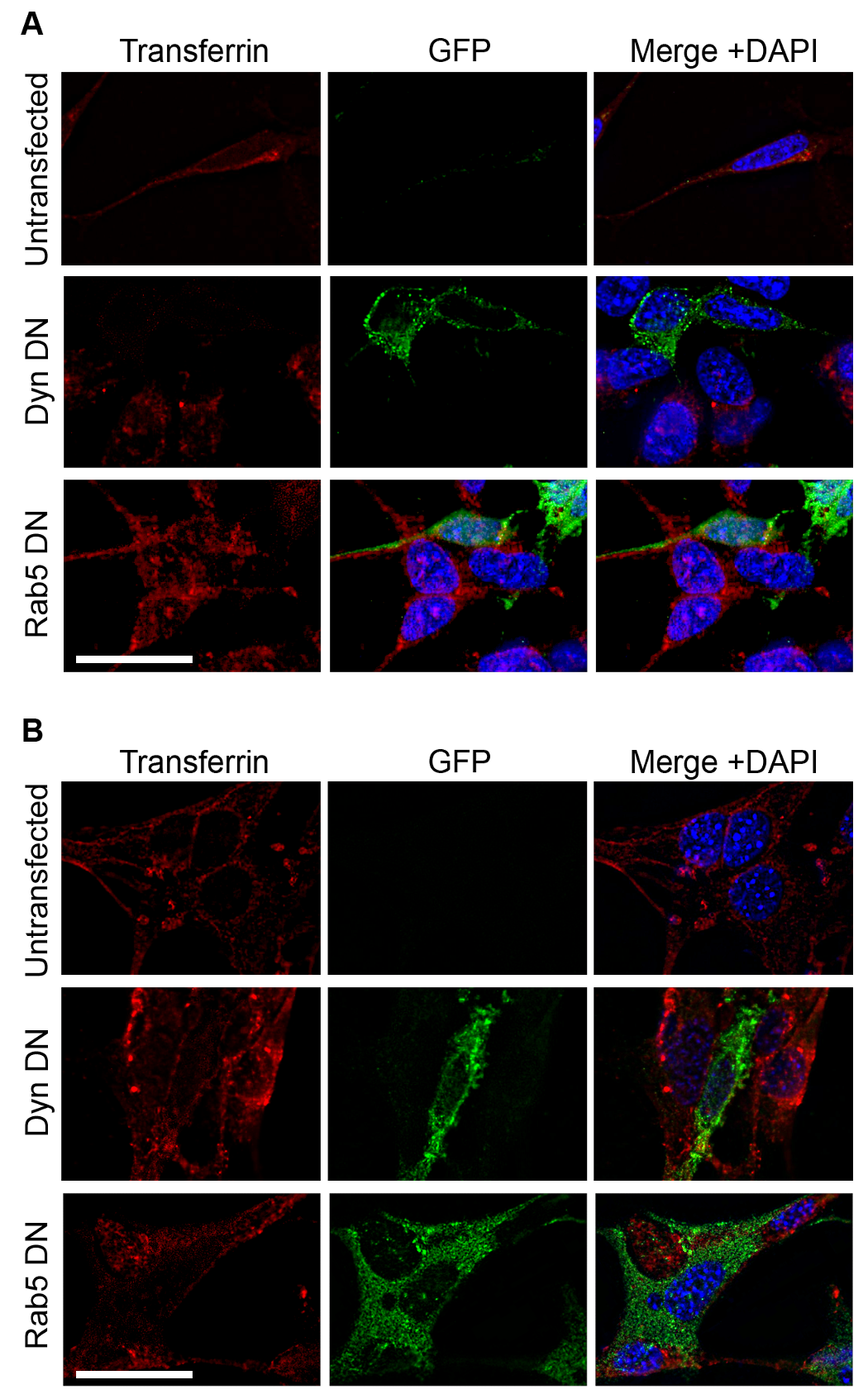

Supplement: S3 Fig — Robust uptake of Alexa 594 labelled transferrin can be seen in both untransfected SH-SY5Y (A) and C8-D1A (B). Transient transfection with either GFP-tagged dominant negative Dynamin1 (Dyn DN) or dominant negative Rab5 (Rab5 DN) prevents transferrin uptake. Scale bars 10μm. (TIF) [file pone.0193302.s003.tif]
